# Supplementary material for: Trophic evolution in ornithopod dinosaurs revealed by dental wear
Source: Nat Commun. 2024 Aug 26;15:7330. doi: 10.1038/s41467-024-51697-9 (PMC11347701; doi:10.1038/s41467-024-51697-9)
Supplement: Supplementary file 1 — Supplementary Information [file 41467_2024_51697_MOESM1_ESM.pdf]

Supplementary information for the manuscript

Trophic evolution in ornithopod dinosaurs revealed by dental wear

by

Attila Ősi*[1, 2], Paul M. Barrett[3], András Lajos Nagy[4], Imre Szenti[5], Lívia Vásárhelyi[5], János Magyar[1, 2], Martin Segesdi[1, 2], Zoltán Csiki-Sava[6], Gábor Botfalvai[1, 7], Viviána Jó[8]

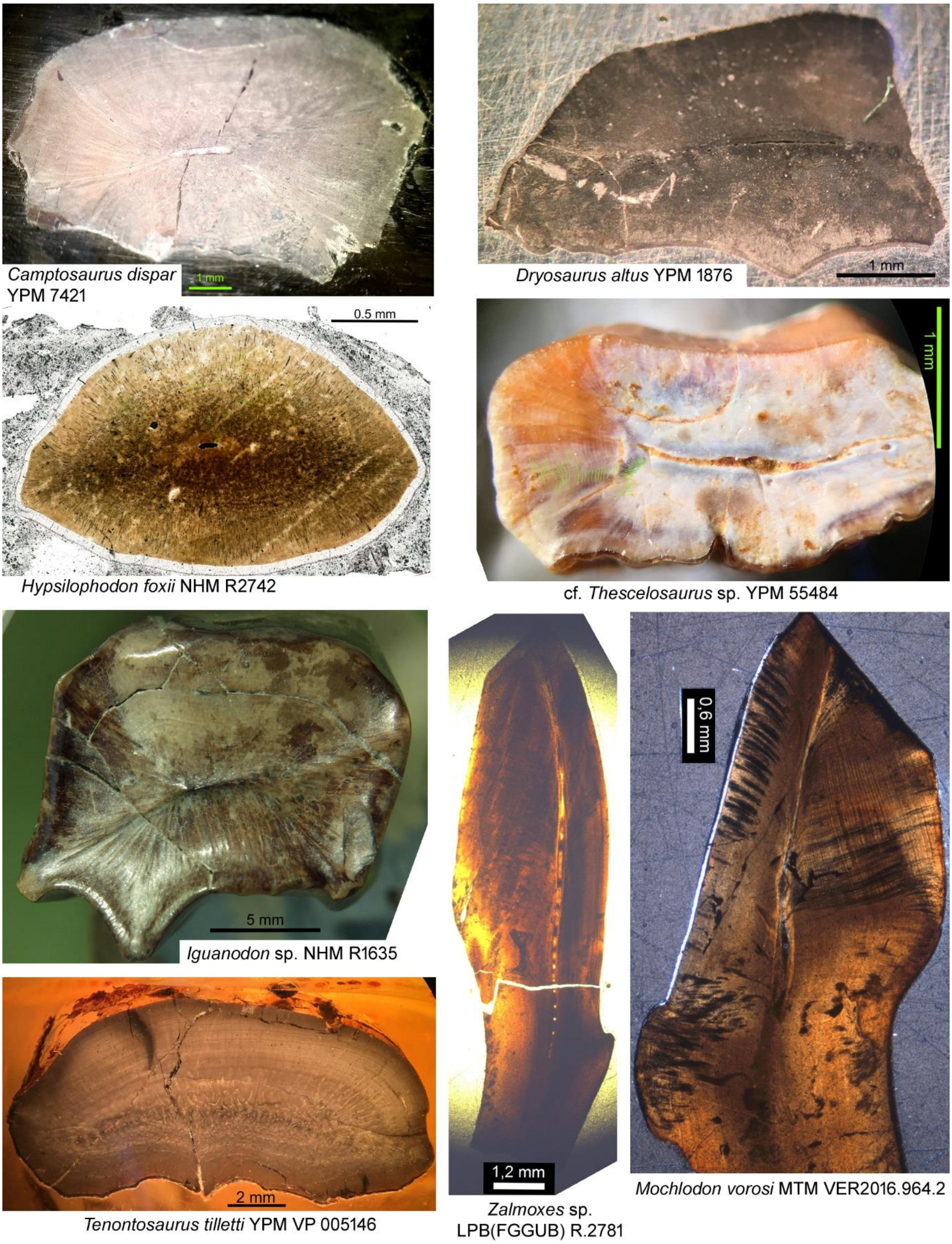

Supplementary figure 1. Horizontal (*Camptosaurus, Dryosaurus, Hypsilophodon, Tenontosaurus*) and labiolingual (*Zalmoxes, Mochlodon*) sections, and occlusal wear surfaces (cf. *Thescelosaurus, Iguanodon*) from the teeth of non-ornithopod genasaurians and non-hadrosaurid ornithopods. On these teeth the minimum number of VELs can be counted and the mean VEIW can be measured.

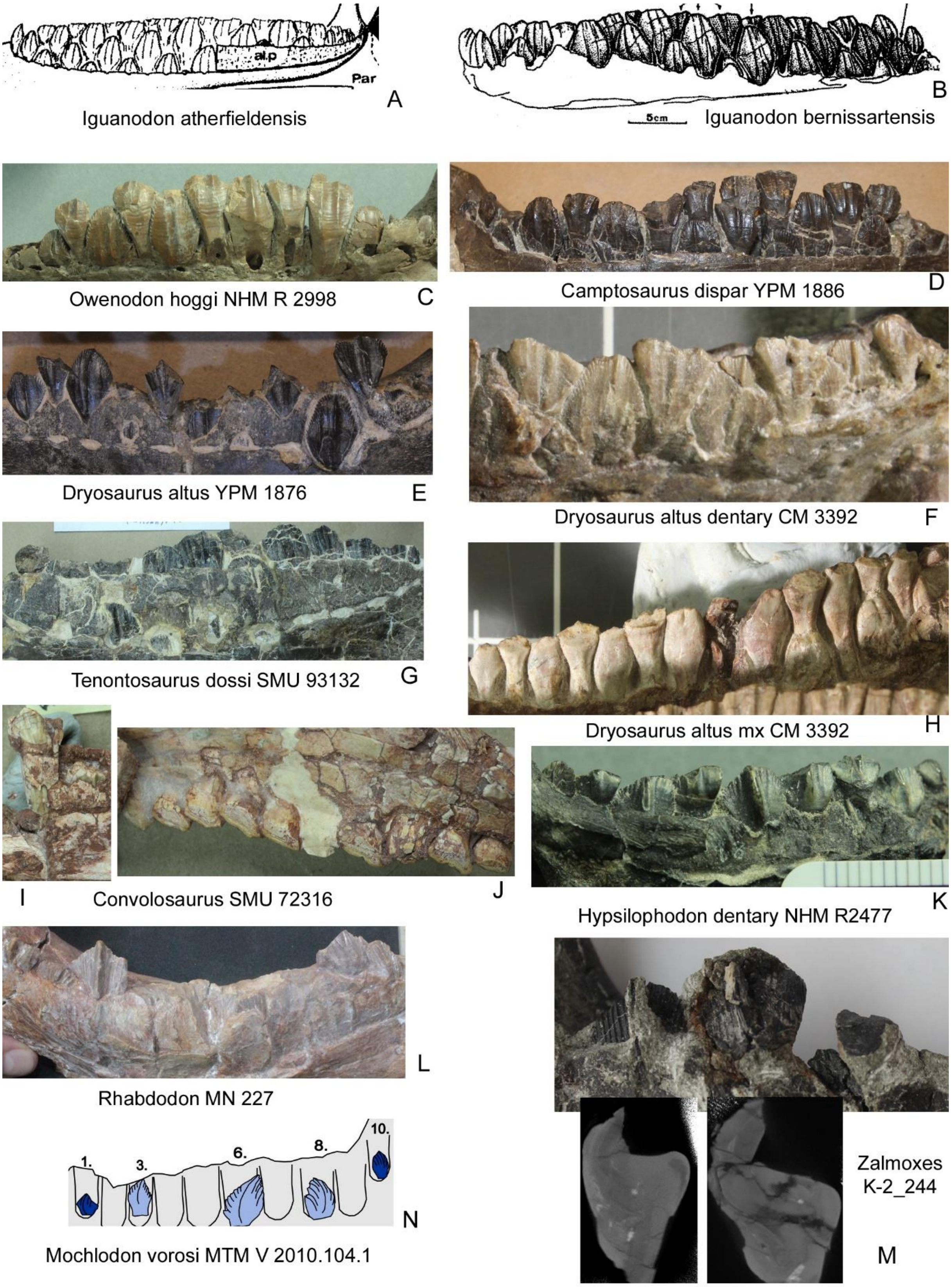

Supplementary figure 2. Dentary dentition in *Iguanodon* (A after Norman 1986, B after Norman 1980), *Owenodon* (C), *Camptosaurus* (D), *Dryosaurus* (E, F), *Tenontosaurus* (G), *Convolosaurus* (I), *Hypsilophodon* (K), *Rhabdodon* (L), *Zalmoxes* (M) and *Mochlodon* (N). Maxillary dentition in *Dryosaurus* (H) and *Convolosaurus* (J).

Supplementary tables on the original specimens studied and where they are housed, including curator names and contact details.

1. List of taxa used for measuring the volumetric data. Institutional abbreviations and curators of the different institutions:

- MTM, Hungarian Natural History Museum
- MAFI, Supervisory Authority for Regulatory Affairs, Geological Survey, Department of
- Collections.
- NHM, Natural History Museum, London, UK, Susannah Maidment
- YPM, Peabody Museum, Yale University, New Haven, USA, Hannah Keller and Daniel Brinkman
- CMNH, Carnegie Museum of Natural History, Pittsburgh, USA, Matthew Lamanna and Amy Henrici
- USNM, Smithsonian National Museum of Natural History, Washington, USA, Matthew Carrano and Hans-Dieter Sues
- SMU, Shuler Museum of Paleontology, Dallas, USA, Louis Jacobs and Dale Winkler
- MN, Musée de Cruzy, Cruzy, France, Didier Clavel
- Velaux, Musée du Moulin Seigneurial, Velaux, France, Xavier Valentin
- AMNH, American Museum of Natural History, New York, USA, Alana Gishlick and Meng Jin
- OUMNH/OXFUM Oxford University, Museum of Natural History, Hilary Ketchum
- Mechin, Patrick and Annie Mechin Collection, Vitrolles, France, Patrick and Annie Mechin
- LPB (FGGUB), University of Bucharest, Vertebrate Paleontological Collection, Zoltán Csiki-Sava
- GPIT-PV, Universität Tübingen, Tübingen, Germany, Felix Augustin and Henrik Stöhr

Taxon	Institute/location	Catalogue number
Zalmoxes	MAFI	**V.13514b**
	MTM	exhibition_1_lower
	MTM	exhibition_2_upper
	MTM	exhibition_3_lower
	MTM	R.2747_upper
	MAFI	V.13514a
	MAFI	V.13514c
	MAFI	V.13531a
	MAFI	V.13514d

	MAFI	V.13514e_upper
	MAFI	Ob_3073_A
Iguanodon	London	**NHM_R364**
	London	NHM_R36500
	London	NHM_R6756
	London	NHM_R3428
	London	NHM_R2394
	London	NHM_R4700
	London	NHM_R1895
	London	NHM_R1635
	London	NHM_R604
	London	NHM_R2387
Matheronodon	Velaux	**mx_tooth_complete**
	Velaux	mx_tooth_worn
Tenontosaurus	USA/AMNH	**AMNH3014**
	USA/AMNH	AMNH3034
	USA/AMNH	AMNH33699
	USA/YPM	YPM5456_associated_tooth_3
	USA/YPM	YPM5456_associated_tooth_4
	USA/YPM	YPM5456_associated_tooth_5
	USA/YPM	YPM5456_associated_tooth_6
	USA/YPM	YPM5456_associated_tooth_8
	USA/YPM	YPM5456_associated_tooth_9
	USA/SMU	SMU76268
	USA/SMU	SMU76267
Thescelosaurus	USA/YPM	**YPM55487**
	USA/CMNH	CM90336
	USA/USMN	PAL427737
	USA/YPM	YPM8537
	USA/YPM	YPM55488
	USA/YPM	YPM55489
Hypsilophodon	London	**NHM_R8367**
	London	NHM_R5863 (tooth in dentary)
	London	NHM_R8418
	London	NHM_R5863 (broken tooth)
	London	NHM_R192
	London	NHM_R2477
	London	NHM_R6372
**Hypsilophodontid (USA-YPM)**	USA/YPM	YPM55478
	USA/YPM	YPM55480
	USA/YPM	YPM55484
Owenodon	London	**NHM_R2998**

	London	NHM_R2998
	London	NHM_R2998
	London	NHM_R2998

Convolosaurus	USA/SMU	**SMU72316**
	USA/SMU	SMU72316_isolated_worn_dentary_tooth_a
	USA/SMU	SMU72316_isolated_worn_dentary_tooth_b
	USA/SMU	SMU72316_izisolated_worn_dentary_tooth_c
	USA/SMU	SMU72316_isolated_worn_dentary_tooth_d
	USA/SMU	SMU_I_AZI

Rhabdodon	Mechin	**554**
	Mechin	797
	Mechin	724
	Cruzy	M269
	Cruzy	M26
	Cruzy	**MN227**
	Cruzy	MN227
	Cruzy	MN227
	Mechin	506
	Mechin	508
	Cruzy	MN116

Cumnoria	OUMNH	OXFUM **J3303**
	OUMNH	OXFUM J3303
	OUMNH	**OXFUM J3303 (left dentary tooth)**
	OUMNH	OXFUM J3303
	OUMNH	OXFUM J3303

Dryosaurus	USA/YPM	**YPM1876**
	USA/YPM	YPM1876
	USA/YPM	YPM1876
	USA/CMNH	CM3392
	USA/CMNH	CM3392
	USA/CMNH	CM3392

Nanosaurus	USA/YPM	**YPM9524**
	USA/YPM	YPM9523

Camptosaurus	USA/USNM	**USNM4697**
	USA/USNM	USNM_V5818
	USA/USNM	USNM_V5818
	USA/USNM	USNM_V7081
	USA/USNM	USNM_V5819
	USA/USNM	USNM_V5819
	USA/YPM	YPM1880
	USA/YPM	YPM1880

Bugenasaura	USA/YPM	YPM8098

Laosaurus	USA/YPM	**YPM1882**
	USA/YPM	**YPM1882**
	USA/YPM	**YPM1882**

Othnielosaurus	USA/USNM	**USNM_V5829**
	USA/USNM	USNM_V5829
	USA/USNM	USNM_PAL412491
	USA/YPM	YPM5748
	USA/YPM	YPM7451

2. List of specimens used for microwear studies.

Taxon	Specimen	Catalogue number
Camptosaurus	1	USNM5818
	2	USNM5818
	3	USNM7081
	4	YMP7416
	5	YPM1880
	6	YPM1880
	7	YPM1886
	8	YPM1886
	9	YPM1886
	10	YPM1886
	11	YPM1886
	12	YPM1886
	13	YPM7416
	14	YPM7416
	15	USNM5819
	16	USNM5819
Convolosaurus	17	SMU.72316
	18	SMU.I-AZI
	19	SMU.72316
	20	SMU.72316
	21	SMU.72316
	22	SMU.72316
	23	SMU.72316
	24	SMU.72316
	25	SMU.72316
Cumnoria	26	OXFUM1.3303
	27	OXFUM1.3303
	28	OXFUM1.3303

Dryosaurus	29	CM3392
	30	CM3392
	31	CM3392
	32	CM3392
	33	CM3392
	34	CM3392
	35	CM3392
	36	CM3392
	37	CM3392
	38	CM3392
	39	CM3392
	40	CM3392
	41	CM3392
	42	CM3392
	43	YPM1876
	44	YPM1876
Hypsilophodon	45	NHMR192
	46	NHMR192
	47	NHMR2472
	48	NHMR5863
	49	NHMR5863
	50	NHMR8367
	51	NHMR8367
	52	NHMR2471
	53	NHMR2471
	54	NHMR2477
	55	NHMR2477
	56	NHMR2477
	57	NHMR2477
	58	NHMR6372
	59	NHMR6372
	60	NHMR8418
Iguanodon	61	R1709
	62	R1709
	63	R1709
	64	R1709
	65	R1709
	66	NHMR6756
	67	NHMR1635
	68	NHMR1895
	69	NHMR2382
	70	NHMR2387
	71	NHMR2387
	72	NHMR3428
	73	NHMR36500
	74	NHMR4700

	75	NHMR604
	76	NHMR754
Mochlodon vorosi	77	MTM 2016.1601a
	78	MTM 2016.1601b
	79	MTM 2016.962
	80	MTM PAL 2022.15.1.
	81	MTM PAL 2022.16.1.
Owenodon	82	NHMR2998
	83	NHMR2998
	84	NHMR2998
	85	NHMR2998
Rhabdodon	86	MN166
	87	MN227
	88	Mechin_No.504
	89	Mechin_No.506
	90	Mechin_No.724
	91	Mechin_No.508
	92	Mechin_No.655
Tenontosaurus dossi	93	SMU_2
	94	SMU.76268
	95	SMU.76267
	96	SMU.76269
Tenontosaurus tilleti	97	YPM5456
	98	YPM5456
	99	YPM5456
	100	YPM5456
	101	YPM5456
	102	YPM5456
	103	YPM5456
Thescelosaurus	104	CM.90336
	105	YPM55488
	106	YPM55489
	107	YPM57177
	108	YPM7435
Zalmoxes	109	LPB (FGGUB) R.2783
	110	LPB (FGGUB) R.2781
	111	LPB (FGGUB) R.2839
	112	LPB (FGGUB) R.2860
	113	LPB (FGGUB) R.2861
	114	LPB (FGGUB) Non catalogised
	115	LPB (FGGUB) R.2747
	116	LPB (FGGUB) R.2782
	117	LPB (FGGUB) R.2838
Edmontosaurus	118	CM12101
	119	CM12101
	120	CM12101

	121	YPM3273
	122	YPM3273
	123	YPM3273
	124	YPM3273
	125	YPM3273
	126	USNM4737
	127	USNM4737
	128	USNM4737
	129	USNM4737
	130	USNM4737
	131	USNM4737
	132	USNM4737
	133	USNM4737
	134	USNM4737
	135	USNMV4808
	136	USNMV4808
	137	USNMV4808
	138	USNMV4808
	139	USNMV4808
Maisaura	140	YPM22400
	141	YPM22400
	142	YPM22400
	143	YPM22400
Dysalotosaurus	144	GPIT-PV 1713-22
	145	GPIT-PV 1713-24
	146	GPIT-PV 1713-25
	147	GPIT-PV 1713-29
	148	GPIT-PV 69062
	149	GPIT-PV 69069
	150	GPIT-PV 72391
	151	GPIT-PV 72391
