# Supplementary material for: Trophic evolution in ornithopod dinosaurs revealed by dental wear
Source: Nat Commun. 2024 Aug 26;15:7330. doi: 10.1038/s41467-024-51697-9 (PMC11347701; doi:10.1038/s41467-024-51697-9)
Supplement: Supplementary file 3 — Description of Additional Supplementary Files [file 41467_2024_51697_MOESM3_ESM.pdf]

Description of Additional Supplementary Files

Tabs and description

Supplementary Data 1

Crown and replacement data: *Tooth crown and replacement rate data for non-onithopod genasaurians and non-hadrosaurid ornithopods.*

Correlation and significance: *Correlation and significance tooth crown and replacement rate data for non-onithopod genasaurians and non-hadrosaurid ornithopods.*

Supplementary Data 2

Crown volume data: *Tooth crown volume data of complete and worn teeth of non-onithopod genasaurians and non-hadrosaurid ornithopods.*

Supplementary Data 3

2D data: *2D microwear data from the wear facets of non-onithopod genasaurians and non-hadrosaurid ornithopods.*

2D data for R: *2D microwear data for R from the wear facets of non-onithopod genasaurians and non-hadrosaurid ornithopods.*

2D PCA results : *2D microwear data PCA results from the wear facets of non-onithopod genasaurians and non-hadrosaurid ornithopods.*

3D DMTA data: *3D microwear DMTA data from the wear facets of non-onithopod genasaurians and non-hadrosaurid ornithopods.*

3D data for R: *3D microwear data for R from the wear facets of non-onithopod genasaurians and non-hadrosaurid ornithopods.*

3D PCA results: 3*D microwear data PCA results from the wear facets of non-onithopod genasaurians and non-hadrosaurid ornithopods.*
