# Supplementary material for: Trophic evolution in ornithopod dinosaurs revealed by dental wear
Source: Nat Commun. 2024 Aug 26;15:7330. doi: 10.1038/s41467-024-51697-9 (PMC11347701; doi:10.1038/s41467-024-51697-9)
Supplement: Supplementary file 7 — Reporting Summary [file 41467_2024_51697_MOESM7_ESM.pdf]

nature portfolio

Corresponding author(s): Attila Ősi

Last updated by author(s): Aug 1, 2024

Reporting Summary

Nature Portfolio wishes to improve the reproducibility of the work that we publish. This form provides structure for consistency and transparency in reporting. For further information on Nature Portfolio policies, see our Editorial Policies and the Editorial Policy Checklist.

Statistics

For all statistical analyses, confirm that the following items are present in the figure legend, table legend, main text, or Methods section.

n/a	Confirmed	
☐	☒	The exact sample size (*n*) for each experimental group/condition, given as a discrete number and unit of measurement
☐	☒	A statement on whether measurements were taken from distinct samples or whether the same sample was measured repeatedly
☐	☒	The statistical test(s) used AND whether they are one- or two-sided<br>*Only common tests should be described solely by name; describe more complex techniques in the Methods section.*
☒	☐	A description of all covariates tested
☒	☐	A description of any assumptions or corrections, such as tests of normality and adjustment for multiple comparisons
☐	☒	A full description of the statistical parameters including central tendency (e.g. means) or other basic estimates (e.g. regression coefficient) AND variation (e.g. standard deviation) or associated estimates of uncertainty (e.g. confidence intervals)
☐	☒	For null hypothesis testing, the test statistic (e.g. *F*, *t*, *r*) with confidence intervals, effect sizes, degrees of freedom and *P* value noted<br>*Give P values as exact values whenever suitable.*
☒	☐	For Bayesian analysis, information on the choice of priors and Markov chain Monte Carlo settings
☒	☐	For hierarchical and complex designs, identification of the appropriate level for tests and full reporting of outcomes
☒	☐	Estimates of effect sizes (e.g. Cohen's *d*, Pearson's *r*), indicating how they were calculated

Our web collection on statistics for biologists contains articles on many of the points above.

Software and code

Policy information about availability of computer code

Data collection	After 3D scanning of the dinosaur tooth specimens the point clouds from the multidirectional scans were merged and surfaces created in FlexScan3D v. 3.3.21.8. Measurements of worn crown surface area and crown volume, were performed in Geomagic Wrap v. 2017.0.2.18 (3D Systems, Rock Hill, SC).
Data analysis	Micrographs taken from worn tooth surfaces were analysed using Microware v. 4.0 (University of Arkansas, USA). Principal components analysis (PCA) was conducted in R software (R v. 4.0.5). Plots showing the relationship between different dental parameters was made in Microsoft Excel.

For manuscripts utilizing custom algorithms or software that are central to the research but not yet described in published literature, software must be made available to editors and reviewers. We strongly encourage code deposition in a community repository (e.g. GitHub). See the Nature Portfolio guidelines for submitting code & software for further information.

Data

Policy information about availability of data

All manuscripts must include a data availability statement. This statement should provide the following information, where applicable:

- Accession codes, unique identifiers, or web links for publicly available datasets
- A description of any restrictions on data availability
- For clinical datasets or third party data, please ensure that the statement adheres to our policy

The datasets generated and/or analysed during the current study are available at: https://doi.org/10.5281/zenodo.11092497

Research involving human participants, their data, or biological material

Policy information about studies with human participants or human data. See also policy information about sex, gender (identity/presentation), and sexual orientation and race, ethnicity and racism.

Reporting on sex and gender	*Use the terms sex (biological attribute) and gender (shaped by social and cultural circumstances) carefully in order to avoid confusing both terms. Indicate if findings apply to only one sex or gender; describe whether sex and gender were considered in study design; whether sex and/or gender was determined based on self-reporting or assigned and methods used.* *Provide in the source data disaggregated sex and gender data, where this information has been collected, and if consent has been obtained for sharing of individual-level data; provide overall numbers in this Reporting Summary. Please state if this information has not been collected.* *Report sex- and gender-based analyses where performed, justify reasons for lack of sex- and gender-based analysis.*
Reporting on race, ethnicity, or other socially relevant groupings	*Please specify the socially constructed or socially relevant categorization variable(s) used in your manuscript and explain why they were used. Please note that such variables should not be used as proxies for other socially constructed/relevant variables (for example, race or ethnicity should not be used as a proxy for socioeconomic status).* *Provide clear definitions of the relevant terms used, how they were provided (by the participants/respondents, the researchers, or third parties), and the method(s) used to classify people into the different categories (e.g. self-report, census or administrative data, social media data, etc.)* *Please provide details about how you controlled for confounding variables in your analyses.*
Population characteristics	*Describe the covariate-relevant population characteristics of the human research participants (e.g. age, genotypic information, past and current diagnosis and treatment categories). If you filled out the behavioural & social sciences study design questions and have nothing to add here, write "See above."*
Recruitment	*Describe how participants were recruited. Outline any potential self-selection bias or other biases that may be present and how these are likely to impact results.*
Ethics oversight	*Identify the organization(s) that approved the study protocol.*

Note that full information on the approval of the study protocol must also be provided in the manuscript.

Field-specific reporting

Please select the one below that is the best fit for your research. If you are not sure, read the appropriate sections before making your selection.

☐ Life sciences ☐ Behavioural & social sciences ☒ Ecological, evolutionary & environmental sciences

For a reference copy of the document with all sections, see nature.com/documents/nr-reporting-summary-flat.pdf

Ecological, evolutionary & environmental sciences study design

All studies must disclose on these points even when the disclosure is negative.

Study description	In this work we studied the teeth of Late Jurassic to Late Cretaceous herbivorous dinosaurs to demonstrate the evolution of dental wear pattern using 3D modelling and numerical analyses by measuring tooth replacement rates and volumes of tooth wear, and document changes in dental microwear pattern.
Research sample	We used the original (in situ or associated) teeth of two non-ornithopod genasaurians, 13 non-hadrosaurid ornithopods and two hadrosaurids.
Sampling strategy	We have choosen those non-ornithopod and ornithopod dinosaur teeth for sampling that were available in UK, European and US collections. These samples cover the most important non-hadrosaurian ornithopods which were critical for a better understanding of the evolutionary change in the feeding mode of these herbivores.

Data collection	We contacted the curators of the different Museum collections and after getting access, we went to the collections and scanned the skull, jaw and/or tooth specimens (Viviána Jó and Attila Ősi). In addition where it was allowed, we took high-resolution silicon moulds from the teeth for microwear analysis (Attila Ősi).
Timing and spatial scale	We've done the data collecting in:<br>- Europe (01.08.2020-15.09.2020, 10.10.2021-30.10.2021, 02.07.2023-07.07.2023)<br>- UK (02.05.2022.-18.05.2022)<br>- USA (30.07.2022-05.09.2022)
Data exclusions	Not applicaple.
Reproducibility	Data taken from the 3D models of the teeth and from the 2D and 3D microwear analysis can be reproduced at any time. Silicon moulds from the teeth are available in the Collection of the Eötvös Loránd University. Digital data are available on the Zenodoo link provided in the MS and in the measurements are in the Supplementary files.
Randomization	Samples were allocated into phylogeny-based groups (two non-ornithopod genasaurians, 13 non-hadrosaurid ornithopods and two hadrosaurids).
Blinding	In this study working with fossils specimens blinding was not relevant.

Did the study involve field work? ☒ Yes ☐ No

Field work, collection and transport

Field conditions	Specimens arising from the field work of our research group belong to Mochlodon, Hungarosaurus (filed work in Iharkút, Hungary) and Zalmoxes (field work in Valioara, Transylvania, Romania). These were summer excavations using the classical field methods (search for fossils, preparation, photo documentation, localization measurements).
Location	Iharkút, Veszprém County, Hungary: 47.2373631,17.6364906<br>Valioara, Transylvania, Romania: 45.6166633,22.778027
Access & import/export	In case of Iharkút the research group had a cooperation during excavation with the Hungarian Dinosaur Foundation who owns the dinosaur site.<br>In case of Valioara, a cooperation with the University of Bucharast permitted the excavation.
Disturbance	None.

Reporting for specific materials, systems and methods

We require information from authors about some types of materials, experimental systems and methods used in many studies. Here, indicate whether each material, system or method listed is relevant to your study. If you are not sure if a list item applies to your research, read the appropriate section before selecting a response.

Materials & experimental systems

n/a	Involved in the study	
☒	☐	Antibodies
☒	☐	Eukaryotic cell lines
☐	☒	Palaeontology and archaeology
☒	☐	Animals and other organisms
☒	☐	Clinical data
☒	☐	Dual use research of concern
☒	☐	Plants

Methods

n/a	Involved in the study	
☒	☐	ChIP-seq
☒	☐	Flow cytometry
☒	☐	MRI-based neuroimaging

Palaeontology and Archaeology

Specimen provenance	- Susannah Maidment (Natural History Museum, London, UK), 20.04.2022<br>- Hannah Keller and Daniel Brinkman (Peabody Museum, Yale University, New Haven, USA), 03.07.2022<br>- Matthew Lamanna and Amy Henrici (Carnegie Museum of Natural History, Pittsburgh, USA), 25.07.2022<br>- Matthew Carrano and Hans-Dieter Sues (Smithsonian National Museum of Natural History, Washington, USA), 15.07.2022<br>- Louis Jacobs and Dale Winkler (Shuler Museum of Paleontology, Dallas, USA), 03.07.2022<br>- Annelise Folie and Pascal Godefroit (Muséum des Sciences naturelles de Belgique, Bruxelles, Belgium), 05.10.2021<br>- Eric Buffetaut (CNRS, Paris, France) and Didier Clavel (Musée de Cruzy, Cruzy, France), 21.10.2021<br>- Patrick and Annie Mechin (Vitrolles, France), 20.10.2021<br>- Oliver Rauhut (Bayerische Staatssammlung für Paläontologie und Geologie, Munich, Germany), 20.06.2023<br>- Felix Augustin and Henrik Stöhr (Universität Tübingen, Tübingen, Germany), 22.06.2023<br>- Xavier Valentin (Musée du Moulin Seigneurial, Velaux, France), 05.10.2021<br>- Alana Gishlick and Meng Jin (American Museum of Natural History, New York, USA), 30.07.2022<br>All permissions were provided via email from the Musuem curators.
Specimen deposition	Specimens from Iharkút collected by our research group are deposited in the Hungarian Natural History Museum.<br>Specimens from Valioara collected by our research group are deposited in the University of Bucharest.
Dating methods	Not applicable.

☐ Tick this box to confirm that the raw and calibrated dates are available in the paper or in Supplementary Information.

Ethics oversight	*Identify the organization(s) that approved or provided guidance on the study protocol, OR state that no ethical approval or guidance was required and explain why not.*

Note that full information on the approval of the study protocol must also be provided in the manuscript.

Plants

Seed stocks	*Report on the source of all seed stocks or other plant material used. If applicable, state the seed stock centre and catalogue number. If plant specimens were collected from the field, describe the collection location, date and sampling procedures.*
Novel plant genotypes	*Describe the methods by which all novel plant genotypes were produced. This includes those generated by transgenic approaches, gene editing, chemical/radiation-based mutagenesis and hybridization. For transgenic lines, describe the transformation method, the number of independent lines analyzed and the generation upon which experiments were performed. For gene-edited lines, describe the editor used, the endogenous sequence targeted for editing, the targeting guide RNA sequence (if applicable) and how the editor was applied.*
Authentication	*Describe any authentication procedures for each seed stock used or novel genotype generated. Describe any experiments used to assess the effect of a mutation and, where applicable, how potential secondary effects (e.g. second site T-DNA insertions, mosiacism, off-target gene editing) were examined.*
